# Potential Ophthalmological Application of Extracts Obtained from Tuna Vitreous Humor Using Lactic Acid-Based Deep Eutectic Systems

**DOI:** 10.3390/foods11030342

**Published:** 2022-01-25

**Authors:** Maha M. Abdallah, Inês C. Leonardo, Luna Krstić, Amalia Enríquez-de-Salamanca, Yolanda Diebold, María J. González-García, Frédéric B. Gaspar, Ana A. Matias, Maria Rosário Bronze, Naiara Fernández

**Affiliations:** 1 Instituto de Tecnologia Química e Biológica António Xavier, Universidade Nova de Lisboa, Avenida da República, Estação Agronómica Nacional, 2780-157 Oeiras, Portugal; maha.abdallah@ibet.pt (M.M.A.); ines.leonardo@ibet.pt (I.C.L.); fgaspar@ibet.pt (F.B.G.); mbronze@ibet.pt (M.R.B.); 2 Instituto de Biologia Experimental e Tecnológica, Avenida da República, Quinta-do-Marquês, Estação Agronómica Nacional, Apartado 12, 2781-901 Oeiras, Portugal; amatias@ibet.pt; 3 Institute of Applied Ophthalmobiology (IOBA), University of Valladolid, 47011 Valladolid, Spain; lkrstic@ioba.med.uva.es (L.K.); amalia@ioba.med.uva.es (A.E.-d.-S.); yol@ioba.med.uva.es (Y.D.); mjgonzalez.ioba@gmail.com (M.J.G.-G.); 4 Biomedical Research Networking Center in Bioengineering, Biomaterials and Nanomedicine (CIBER-BBN), 28029 Madrid, Spain; 5 Faculty of Pharmacy, University of Lisbon, Av. Prof. Gama Pinto, 1649-003 Lisboa, Portugal

**Keywords:** food waste valorization, natural hyaluronic acid, deep eutectic solvents, ocular therapy, antioxidant, anti-inflammatory, antimicrobial

## Abstract

A green technique was developed to extract hyaluronic acid (HA) from tuna vitreous humor (TVH) for its potential application in managing dry eye disease. Deep eutectic solvents (DES) were used to extract HA and were synthesized using natural compounds (lactic acid, fructose, and urea). The DES, the soluble fraction of TVH in DES (SF), and the precipitated extracts (PE) were evaluated for their potential use in dry eye disease treatment. In vitro experiments on human corneal epithelial cell lines and the effect on dry eye-associated microorganisms were performed. The influence of the samples on the HCE viability, their intracellular reactive oxygen species (ROS) scavenging capacity, inflammatory response, and antimicrobial properties were studied. According to the results, all samples displayed an antioxidant effect, which was significantly higher for PE in comparison to SF. Most of the tested samples did not induce an inflammatory response in cells, which confirmed the safety in ophthalmic formulations. In addition, the DES and SF proved to be efficient against the studied bacterial strains, while PE did not show an antimicrobial effect. Hence, both DES and SF at defined concentrations could be used as potential compounds in dry eye disease management.

## 1. Introduction

Nowadays, seeking ingredients from natural sources is on the rise as companies are more committed to the sustainable development of green products [1]. The use of marine by-products as a source of natural ingredients has attracted great attention due to their abundance, low cost, safety, and environmental benefits [2]. The total mass of world marine products obtained from fisheries and aquaculture was estimated to be 170.9 million tons in 2016 compared to 151.2 million tons of human consumption [3]. Thus, a significant amount of marine waste is generated annually, commonly discarded on land or in the sea, contaminating the coastal water and air [4]. Therefore, the use of marine waste as a source for the extraction of natural compounds has great advantages in waste valorization and natural ingredients isolation. The Food and Agricultural Organization refers to food waste as the disposal of inedible food fractions along the entire food production and distribution chain [5]. Some of these wastes are classified as avoidable food waste, depending on the causes that lead to its generation, which could be prevented. Hence, using inedible food wastes as a source of bioactive molecules for drug formulation is of high value with environmental and therapeutical benefits.

There are various techniques for efficiently extracting materials from biomass sources, including the use of novel deep eutectic solvents (DES) [6]. These solvents are formed by combining hydrogen bond donor and acceptor molecules and are characterized by having a melting point lower than that of the compounds used to prepare them [7]. These have been shown to be promising molecular solvents due to their advantageous tunability, which allows the optimization of their solubilizing capacity and viscosity, among other different physicochemical properties of interest depending on their application [8,9]. Previous studies and ongoing research have shown various potential uses of versatile combinations of DES in pharmaceutical, biochemical, and other industrial applications [10]. One of the most important aspects of DES is their multifunctional role in the solubilization, extraction, purification, and production of valuable products from biomass [11,12].

In this work, DES were prepared using lactic acid, fructose, and urea to extract hyaluronic acid (HA) from the marine raw material tuna vitreous humor (TVH). The use of DES replaces the time-consuming and/or conventional toxic methods to extract natural HA. Various techniques have been applied to extract HA from natural sources, such as terrestrial and marine biomass [6,13,14]. HA is a hydrophilic molecule with an important role in preserving the hydration and the elastoviscosity of tissues, such as the vitreous humor and the synovial fluid, and in lubricating numerous moving parts, such as the muscles and the joints [15]. Its non-immunogenic and biocompatible effects have significantly increased its application in the pharmaceutical and medical fields, including joint injections, osteoarthritis treatment, ocular therapy, plastic surgeries, and skin treatments [16]. In recent years, HA has been increasingly used in formulations for patients suffering from dry eye disease, as it has been shown to improve the corneal epithelial barrier and the tear film stability [17,18,19,20]. Dry eye is a highly prevalent inflammatory disease in which ocular surface epithelia and the tear film are altered, leading to visual impairment, discomfort, eye dryness, burning, and pain [21,22,23]. Tear production stimulation and tear replacement agents have been used for symptom relief, including HA-based artificial tears, which restore the homeostasis of the tear film [18,24]. Hence, the biocompatibility of DES and HA-containing samples employed in this work were studied on human corneal epithelial cells to evaluate their potential use in ophthalmic formulations to treat dry eye disease. In addition, the antimicrobial effect of the samples was tested using dry eye-associated bacterial species, such as *Staphylococcus aureus* and *Pseudomonas aeruginosa,* to assess whether the samples may also reduce bacterial growth, which would be helpful in the management of dry eye cases prone to infections.

## 2. Materials and Methods

### 2.1. Materials

The following reagents were used in the extraction and chemical characterization processes. Ethanol absolute (≥99.9%, CAS: 64-17-5, Carlo Erba Reagents, Val-de-Reuil, Normandie, France), hyaluronic acid disaccharide (di-HA, CAS: 149368-06-9, Santa Cruz Biotechnology, Inc., Dallas, TX, USA), DL-lactic acid (aqueous solution, 85.0–90.0%, CAS: 50-21-5, Alfa Aesar, Tewksbury, MA, USA), sodium phosphate (96%, CAS: 7601-54-9, Sigma-Aldrich, Burlington, MA, USA. The reagents D-fructose (CAS: 57-48-7), urea (CAS: 57-13-6), Chondroitinase ABC from *Proteus vulgaris* (CAS: 9024-13-9), and sodium chloride (≥99%, CAS: 7647-14-5) were obtained from Sigma-Aldrich (Burlington, MA, USA).

The following reagents were used in cell-based studies. Penicillin-streptomycin, insulin (CAS: 11061-68-0), epidermal growth factor (EGF, E9644, CAS: 62253-63-8), benzalkonium chloride (8001-54-5), and phenazine methosulfate (PMS, CAS: 299-11-6) were obtained from Sigma-Aldrich (Burlington, MA, USA). Dulbecco’s Modified Eagle Medium (DMEM)/F-12 + GlutaMAX-I, fetal bovine serum, tetrazolium salt (2,3-bis-(2-methoxy-4-nitro-5-sulfophenyl)-2H-tetrazolium-5-carboxanilide, XTT, (CAS: 298-96-4), the BCA protein kit, and DMEM/F12 without phenol red were obtained from Thermo Fischer Scientific (Waltham, MA, USA). Cell permeant 2′,7′-dichlorodihydrofluorescein diacetate (H_2_DCF-DA) dye (CAS: 4091-99-0) was obtained from Merck Life Sciences (Darmstadt, Germany). Tumor necrosis factor-α (TNF-α, CAS: 94948-59-1) and the IL-6 (REF 950.030.192) and IL-10 (REF 950.060.192) ELISA kits (Diaclone) were obtained from bioNOVA cientifica s. l. (Madrid, Spain).

The following reagents were used in the microbial studies. Cation-adjusted Mueller Hinton broth (CAMHB) was obtained from BD (Sparks, MD, USA), trypticase soy agar (TSA) and trypticase soy broth (TSB) were obtained from VWR (Monroeville, PA, USA). Gram-positive bacteria *Staphylococcus aureus* ATCC 6538 and gram-negative bacteria *Pseudomonas aeruginosa* ATCC 9027 were selected for the antimicrobial susceptibility testing assays. Two commercially available eye drops were used: HYABAK^®^, with 0.15% HA (Théa Pharmaceuticals, Clermont-Ferrand, France), and Clorocil eye drop, with 8 mg/mL chloramphenicol (Edol Laboratory, Carnaxide, Portugal).

### 2.2. Raw Material Conditioning

The raw material tuna eyes were kindly donated from Tunipex, Faro, Portugal. They were kept frozen and were cut to separate the tuna vitreous humor (TVH) from the eyeball. The frozen vitreous humor was then freeze-dried and stored at −20 °C until needed.

### 2.3. Extraction Process

The DES were synthesized by combining the natural components at specific molar ratios under heating at 80 °C and stirring for 30 min until a homogeneous liquid system was obtained. The systems prepared were lactic acid:fructose (DES_LA:F_) and lactic acid:urea (DES_LA:U_) at a molar ratio of (5:1) and (4:1), respectively. The freeze-dried raw materials were mixed with the prepared solvent systems at a mass ratio of 1:100 (raw materials:DES), and they were stirred for 24 h at 50 °C [25]. Then, the mixtures were centrifuged at 6000 rpm for 15 min at 45 °C to separate the undissolved particles. The supernatant was collected, and it represents the testing samples of the DES with the soluble fraction of TVH (SF_LA:F_ and SF_LA:U_). Precipitation of the dissolved extracts was performed by the addition of 3 times the DES volume of ethanol as an anti-solvent [26]. The precipitate was then collected by centrifugation at 6000 rpm for 15 min at 40 °C and dried at 50 °C for 6 h. The obtained precipitated extracts samples represent the testing samples of the extracts (PE_LA:F_ and PE_LA:U_). The experiments were performed in triplicate.

### 2.4. HA Quantification

The PE samples obtained from the extraction using DES were submitted to enzyme hydrolysis before quantification using high-performance liquid chromatography (HPLC). The long-chain HA was hydrolyzed using the enzyme chondroitinase ABC to obtain its disaccharide unit. The hydrolysis was performed by treating 40 μg of the sample with 25 mU of chondroitinase ABC in a sodium phosphate buffer (1 mM, pH = 7) at 37 °C for 3 h [27]. The enzyme was then inactivated by boiling the mixture for 1 min. The HA disaccharide (di-HA) was quantified using HPLC (Waters Alliance 2695 HPLC System, Waters Chromatography, Milford, MA, USA), equipped with Spherisorb SAX Column, 80 Å, 5 µm, 4.6 mm x 250 mm (Waters Portugal—Codigíndice Unipessoal, Lda.). The absorption was monitored using a Waters Photodiode Array Detector 2996 operated by Empower Pro (version 5, 2002) at 232 nm. First, an isocratic separation (0.05 M sodium chloride, pH 4) was applied for 5 min. A linear gradient was then applied from 5 to 25 min (0.05 to 1.2 M sodium chloride, pH 4), followed by 10 min of isocratic separation (0.05 M sodium chloride, pH 4). The flow rate was set at 1.2 mL/min [26,27]. The calibration curve was obtained using the di-HA standard at concentrations in the range of 2.5 to 40 µg/mL, prepared with two-fold serial dilutions. The HPLC limit of detection was determined experimentally by serial dilution of the standard, and it was found to be 0.125 g/mL of di-HA in the quantified sample. Experiments were performed in triplicate and the HPLC analysis in duplicate.

### 2.5. In Vitro Studies

#### 2.5.1. Cellular Viability

The two lactic acid-based DES and their corresponding SF and PE testing samples were evaluated in vitro on human corneal epithelial (HCE) cell line [28]. The HCE cell line was cultured in DMEM/F-12 + GlutaMAX-I supplemented with 10% fetal bovine serum, 10 ng/mL of EGF, 5 µg/mL of insulin, 100 U/mL of penicillin, and 100 µg/mL of streptomycin. The cells were kept in a 5% CO_2_ atmosphere at 37 °C. The cellular viability was assessed using the XTT-based colorimetric assay. HCE cells were seeded in 96-well plates, starting with the 38th cell passage, and grown to 90% of pre-confluence. After 24 h, the cells were maintained in a non-supplemented and serum-free medium for another 24 h. A stock solution of each compound was prepared in pure cell culture medium and filtered using sterile 0.2 µm filters. The prepared stock solution concentration was 12 mg/mL for the DES samples and 4 mg/mL for the SF and PE samples. Serial dilutions of the tested compounds were performed in the plates. The cells with the treatments were kept for 24 h at 37 °C. Cells that were treated with the culture medium were used as the negative control, and cells treated with benzalkonium chloride (0.005% *w*/*v* dissolved in culture medium) were used as the positive control. To perform the XTT colorimetric assay, 100 µL of DMEM/F12 without phenol red was added to each well, followed by 25 µL of XTT and PMS mixture prepared prior to its use by adding 10 µL of PMS to the 1 mg/mL XTT solution. Incubation was performed at 37 °C for 3 h. The absorbance was then assessed using a UV/Vis microplate multi-reader at 450 nm and 620 nm (SpectraMax M5; Molecular Devices, Sunnyvale, CA, USA). The experiments were performed in triplicate for each condition. The percentage of viable cells in the treated cells in comparison to the control was computed using the following equation:

$$\%\;\mathrm{cell}\;\mathrm{viability}=\frac{{\mathrm{Abs}}_{450\;\mathrm{nm}}\;\mathrm{sample}\;–{\;\mathrm{Abs}}_{620\;\mathrm{nm}}\;\mathrm{sample}}{{\mathrm{Abs}}_{450\;\mathrm{nm}}\;\mathrm{control}\;–{\;\mathrm{Abs}}_{620\;\mathrm{nm}}\;\mathrm{control}}\times 100$$

#### 2.5.2. Antioxidant Effect

The antioxidant effect of the compounds was analyzed using the H_2_DCF-DA dye to measure intracellular reactive oxygen species (ROS) levels in UV-B radiation-exposed cells. This non-fluorescent dye diffuses in the cells and is cleaved into H_2_DCF that is further oxidized to fluorescent DCF by the ROS. The HCE cells were cultured in 24-well plates and grown to 90% of pre-confluence. They were then maintained in a medium that is serum- and supplement-free for 24 h at 37 °C. The cells were then pre-treated with the compounds for 1 h at 37 °C at specific concentrations selected based on the results obtained from Section 3.2. After pre-treatment, the supernatant was discarded, and 500 µL of 10 µM dye was added to the cells, which were incubated for another 30 min at 37 °C. The supernatant was discarded, and the cells were treated with the same treatments as in the pre-treatment step. They were then exposed to 8-W UV-B light for 15 s, with the lamps located 3 cm below the cells, at 302 nm excitation peak and 7.15 mW/cm2 UV-B radiation power density (Bio-Rad, Inc., Hercules, CA, USA). The cells were then incubated for 1 h at 37 °C. The control cells were kept in incubation without UV-B light stimulation. The fluorescence was obtained using a spectrophotometer at 488 nmex/522 nmem (SpectraMax M5; Molecular Devices, Sunnyvale, CA, USA). The obtained data were normalized to the corresponding total protein content in the adherent cells using the BCA protein assay kit. The experiments were performed in triplicate, and the sample treatments were performed in duplicate.

#### 2.5.3. Evaluation of the Potential Inflammatory Response

The potential inflammatory effect of extracted compounds on corneal epithelial cells was evaluated in vitro, measuring cytokine/chemokine secretion after HCE cells stimulation using TNF-α. Similar to previous studies, HCE cells were cultured in 24-well plates and grown to 90% of pre-confluence, then maintained in a serum- and supplement-free medium for 24 h at 37 °C. The cells were then pre-treated for 2 h at 37 °C with the compounds at specific concentrations. Following this, TNF-α at a concentration of 25 ng/mL was added to the cells for 24 h. Cell supernatants were collected after 24 h, and interleukins IL-6 and IL-10 production quantified with human interleukins (IL-6 and IL-10) ELISA kits according to manufacturer’s instructions. Interleukins’ concentration in each well was normalized to total protein content determined by the BCA protein assay kit. The experiments were performed in triplicate, and the samples were performed in duplicate.

#### 2.5.4. Statistical Analysis

The statistical analysis of the biocompatibility studies on the HCE cell line was analyzed using the SPSS software package (SPSS version 15.0 for Windows; SPSS, Inc., Chicago, IL, USA). Results were expressed as mean ± standard error of the mean (SEM). One-way analysis of variance (ANOVA) with Tukey’s post hoc test or Games-Howell test was used for intergroup comparisons. *p* values < 0.05 were considered statistically significant. GraphPad Prism software (GraphPad Software, Inc., La Jolla, San Diego, CA, USA) was used for the figure plotting.

#### 2.5.5. Antimicrobial Activity

The antimicrobial activity studies of the compounds were carried out using *S. aureus* and *P. aeruginosa*. The stock solutions of the DES, SF, and PE testing samples were prepared by dissolving the samples at specific concentrations (shown in Table S1) in CAMHB. All samples were filter sterilized using 0.2 µm filters. The antimicrobial activity of the samples was compared to two commercially available eye drops: an HA-based eye drop (HA-ED) used for ocular hydration and relief, containing 0.15% HA, and the antibiotic chloramphenicol-based eye drop (CHL-ED) used to combat ocular infection, containing 8 mg/mL chloramphenicol.

The assay was performed based on the broth microdilution method according to the CLSI M07-A10 guidelines [29]. In brief, the bacterial suspensions were diluted using CAMHB, and the optical density was standardized at 600 nm using Ultrospec 2100 pro (Biochrom, Holliston, MA, USA) to obtain a density equivalent to the 0.5 McFarland’s standard. Standardized bacterial suspension (50 μL) was used to inoculate a 96-well microtiter plate wells containing 50 μL of a two-fold dilution series range of the testing sample, achieving a final bacterial density per well of 10^6^ CFU/mL. The plates were incubated at a temperature of 37 °C for 16–20 h. Culture medium without any added compounds was used as a negative control, and bacterial culture without the addition of any agent was used as the positive control. The well having the lowest concentration with no visible bacterial growth observed corresponds to the minimum inhibitory concentration (MIC). In addition, the minimum bactericidal concentration (MBC) was determined by plating 100 μL from the wells with no growth on a TSA medium and incubating for 16–24 h at 37 °C. The MBC can be defined as the lowest concentration that killed at least 99.9% of the bacteria over the period of the assay and is complementary to the MIC. Results of the MIC and MBC were expressed as the median value obtained for each of the three replicates. A test was performed using a two-fold serial dilution of the samples with the addition of sterile medium to ensure their sterility.

## 3. Results and Discussion

### 3.1. Extraction Process

DES were used for the extraction of HA from TVH, a natural marine by-product that is abundantly discarded. This method could not only ensure the use of natural HA for a therapeutic purpose but also implement the valorization of the discarded TVH in a green and low-cost process using DES. HA is a highly valuable biopolymer that has shown increased use in pharmaceutical formulations, namely in tear formulations for dry eye treatment [30]. For instance, previous studies have proven that HA eye drops have a better performance than non-HA eye drops, including artificial tears and normal saline [18,31].

**Figure 1:**
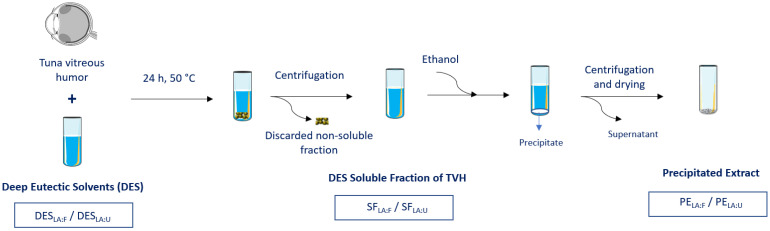
Scheme of the extraction process. Deep eutectic solvents synthesized using lactic acid with fructose and urea are DES_LA:F_ and DES_LA:U_, respectively. The soluble fractions of tuna vitreous humor (TVH) in DES_LA:F_ and DES_LA:U_ are SF_LA:F_ and SF_LA:U_, respectively. Precipitated extracts from DES_LA:F_ and DES_LA:U_ are PE_LA:F_ and PE_LA:U_, respectively.

Preliminary HA solubility studies were performed with different combinations of natural DES previously described in the literature [32,33,34]. Lactic acid-based systems were the most promising in dissolving HA. Therefore, DES were prepared by combining lactic acid with fructose and urea as they are natural, low-cost, and non-toxic compounds that have been applied in several drug formulations and therapeutical applications [35,36,37]. Lactic acid is a carboxylic acid widely employed in formulations due to its biobased and biodegradable features and was already described to enhance the stability and the shelf life of Miconazole eye drops [37,38,39]. On the other hand, fructose is a naturally occurring monosaccharide that has been used in the food and pharmaceutical industries. It has been employed as an excipient in drug formulations due to its safety [36,40]. Also, it was shown to be present in the eye, mainly in the aqueous humor and the stroma, obtained by the sorbitol pathway in the lens [41,42]. Additionally, urea is an endogenous metabolite used in drug formulations for the treatment of skin and ocular diseases, hyponatremia, malignancy, among others [35,43,44,45]. It is a compound formed in ocular tissues and is an important constituent of the tear fluid, as lower urea levels are observed in the tear film of patients with dry eye [46,47]. Considering the relevance and applicability of these compounds, two distinct DES were synthesized in this work combining lactic acid with either fructose or urea (DES_LA:F_ and DES_LA:U_, respectively). Figure 1 displays the scheme of the extraction process and the testing samples and their designations. After mixing DES with TVH, the undissolved part of TVH was discarded to obtain the TVH soluble fraction in DES (SF_LA:F_ and SF_LA:U_), and the extracts were then precipitated (PE_LA:F_ and PE_LA:U_). All samples of the process (DES, SF, and PE) were tested to evaluate their bioactivity. This allowed the evaluation of DES, not only as an extracting solvent but also as part of a therapeutic system containing the soluble fraction of TVH in DES. The comparison between the SF and PE samples was performed to study the differential bioactivity between the compounds. This also allows assessing a possible combined effect of DES with the soluble extracts since enhanced bioactivity, in this case, can eliminate the requirement for extract precipitation from DES, which consequently decreases the process time and cost. The extracts characterization is shown in Table S2 of the Supplementary Materials. The HA amount was quantified using HPLC, and its yield was shown to be 1.9% and 1.1% (mg HA/100 mg extract) in PE_LA:F_ and PE_LA:U_, respectively (Table S2).

### 3.2. Cell Viability

**Figure 2:**
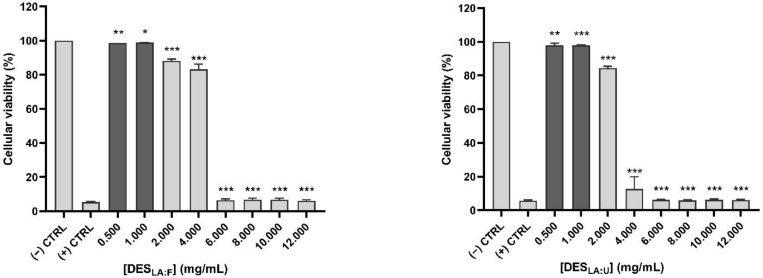

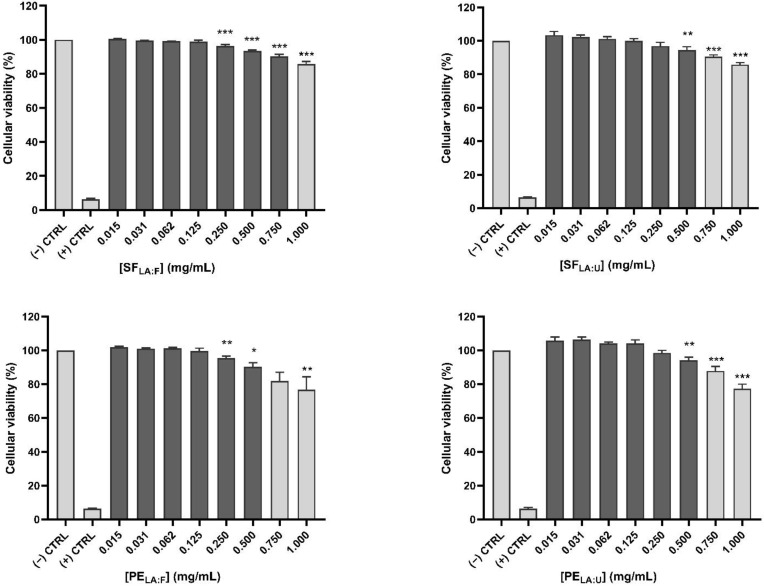
Cellular viability (%) of human corneal epithelial cells after treatment with the testing samples. The concentrations with 90% of cell viability or higher are shown in dark grey bars. Deep eutectic solvents synthesized using lactic acid with fructose and urea are DES_LA:F_ and DES_LA:U_, respectively. The soluble fractions of tuna vitreous humor in DES_LA:F_ and DES_LA:U_ are SF_LA:F_ and SF_LA:U_, respectively. Precipitated extracts from DES_LA:F_ and DES_LA:U_ are PE_LA:F_ and PE_LA:U_, respectively. (* *p* < 0.05, ** *p* < 0.01, *** *p* < 0.001).

The effect of each testing sample on cell viability of HCE cells was studied at different concentrations, as shown in Figure 2. Measurement of cell viability enabled the quantification of live cells numbers after treatment with the samples, which was expressed as a percentage relative to the control. The concentrations with 90% of cell viability or higher (shown in dark grey bars in Figure 2) were considered within an acceptable range [48,49]. These concentrations with higher or equal to 90% viability were selected for the antioxidant and inflammatory responses. The highest viable concentration for both DES was shown to be 1 mg/mL, and they displayed a higher value than that of the SF and PE samples. In addition, the SF samples had a higher or same concentration values in comparison to the PE samples. For instance, the highest viable concentration for SF_LA:F_ was 0.75 mg/mL, greater than that of the PE_LA:F_ (0.5 mg/mL). In contrast, SF_LA:U_ and PE_LA:U_ had the same highest viable concentration of 0.5 mg/mL.

### 3.3. Antioxidant Effect

**Figure 3:**
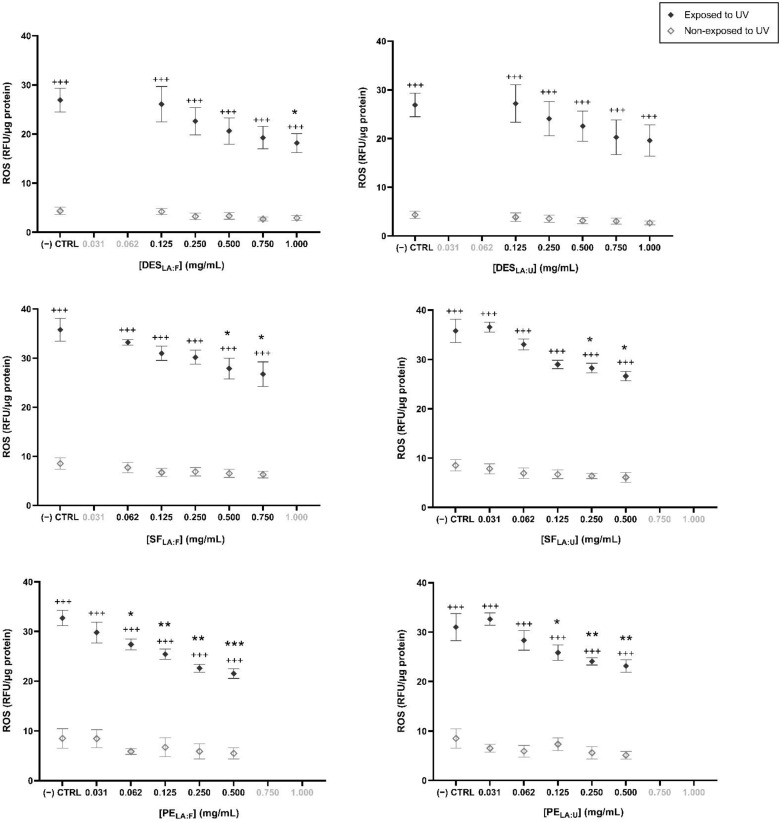
Antioxidant effect of each sample in the human corneal epithelial cell line. Deep eutectic solvents synthesized using lactic acid with fructose and urea are DES_LA:F_ and DES_LA:U_, respectively. The soluble fractions of tuna vitreous humor in DES_LA:F_ and DES_LA:U_ are SF_LA:F_ and SF_LA:U_, respectively. Precipitated extracts from DES_LA:F_ and DES_LA:U_ are PE_LA:F_ and PE_LA:U_, respectively. * *p* < 0.05, ** *p* < 0.01, *** *p* < 0.001 in comparison to UV-stimulated control cells; +++ *p* < 0.001 in comparison to non-UV-stimulated cells.

The antioxidant effect of the samples was evaluated to analyze their potential use in ophthalmic therapy. Previous studies have demonstrated that HA-containing eye drops could decrease oxidative stress and inflammation, which consequently improves dry eye symptoms [50]. An antioxidant effect is observed when the production of intracellular ROS after an external stimulus (UV-B radiation) decreases in comparison to a control upon treatment with a given sample. For most tested samples, a positive antioxidant effect was observed as the production of ROS by HCE cells was decreased in their presence. The ROS production in UV stimulated cells was significantly higher than in non-UV stimulated cells (*p* < 0.001) in the testing samples, as shown in Figure 3. For the DES_LA:F_, the production of ROS in the highest tested concentration (1 mg/mL) significantly decreased in comparison to the control. No antioxidant effect was observed for the studied doses of DES_LA:U_. A significant decrease in ROS production was observed for SF_LA:F_ and SF_LA:U_ in comparison to the control at concentrations of 0.5 and 0.75 mg/mL for SF_LA:F_, and 0.25 and 0.5 mg/mL for SF_LA:U_. Furthermore, PE_LA:F_ and PE_LA:U_ showed antioxidant capacity at different tested concentrations, including low concentrations of 0.062 and 0.125 mg/mL for PE_LA:F_ and PE_LA:U_, respectively. Hence, the precipitation of the bioactive ingredients extracted from TVH displays a significant antioxidant property.

**Figure 4:**
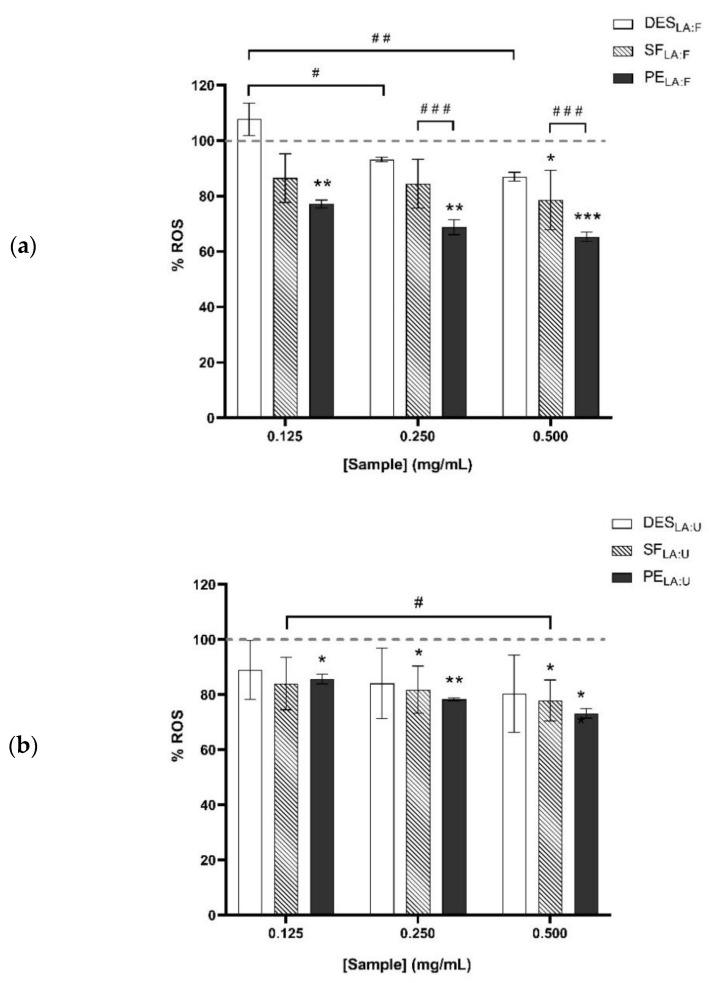
Percentage of ROS in comparison to the control (represented in the dashed line) for the samples of the extraction process obtained (**a**) using deep eutectic solvents synthesized using lactic acid and fructose (DES_LA:F_), and (**b**) using deep eutectic solvents synthesized using lactic acid and urea DES_LA:U_. Their corresponding soluble fractions of TVH in DES_LA:F_ and DES_LA:U_ are SF_LA:F_ and SF_LA:U_, respectively. Their corresponding precipitated extracts from DES_LA:F_ and DES_LA:U_ are PE_LA:F_ and PE_LA:U_, respectively. * *p* < 0.05, ** *p* < 0.01, *** *p* < 0.001 in comparison to UV-stimulated control cells. # *p* < 0.05, ## *p* < 0.01, ### *p* < 0.001 in comparison between the compounds and different doses.

To compare the antioxidant effect between the testing samples, the percentage of detected ROS was quantified in comparison to the control (Figure 4). A higher significant decrease was shown for the PE_LA:F_ sample in comparison to SF_LA:F_. Therefore, the PE_LA:F_ samples are the most promising compounds having a higher significant antioxidant effect.

### 3.4. Evaluation of a Potential Inflammatory Response

Previous studies have demonstrated that low molecular weight HA has pro-inflammatory properties [51,52]. The evaluation of a potential inflammatory response induced by our samples in the corneal cells was performed by measuring interleukins IL-6 and IL-10 levels in cell culture supernatants both in basal, unstimulated, and stimulated cells following an inflammatory stimulus (TNF-α treatment for 24 h). We selected those interleukins considering their reported level alterations in dry eye [53]. When an inflammatory process takes place, IL-10 is secreted as an anti-inflammatory response. IL-6, a pro-inflammatory cytokine, is detected when cell inflammation occurs. As expected, TNF-α-exposed cells secreted significant IL-6 levels, but not IL-10 levels compared to that of control unexposed cells. The tested compounds did not influence the IL-10 secretion, as it was not detected in any sample (Figure S1). In addition, none of the compounds tested significantly increased basal IL-6 secretion by the cells, except for the case of SF_LA:F_ at 0.5 mg/mL (Figure S2). Therefore, all the other doses and compounds did not exhibit a pro-inflammatory behavior in our experimental conditions. Hence, this could potentially prove that these testing samples are safe ingredients for ocular drug formulations without eliciting a pro-inflammatory response.

### 3.5. Antimicrobial Activity

Dry eye disease caused by tear deficiency or excessive tear evaporation conditions is often associated with ocular surface conditions, such as meibomian gland dysfunction, anterior blepharitis, keratitis, among others. These conditions lead to modifications in the ocular surface in the type and concentration of bacteria, including *S. aureus* and *P. aeruginosa* [54]. Hence, the antimicrobial potential of the samples was tested against these two bacterial species to assess if the samples inhibit bacterial growth for dry eyes that are prone to infections. In addition, an antimicrobial activity could lead to the use of the samples as preservatives to ensure a safe shelf life increase of the formulation. The concentrations of the testing samples were analyzed with a two-fold dilution series, having a range of 0.03 to 16 mg of sample/mL for DES and SF and 0.002 to 1 mg of sample/mL for PE. For the PE samples, the highest assessed concentration was lower due to the limited solubility of the extract in the medium. The testing samples were compared to two commercially available eye drops, HA-ED and CHL-ED. The MIC and MBC median values against *S. aureus* and *P. aeruginosa* are shown in Table 1, and the values for the three replicates are shown in Table S3.

It was possible to observe that SF samples presented a similar range of MIC and MBC values in comparison to DES. Hence, the presence of the TVH soluble fraction in DES did not improve its antimicrobial effect remarkably. A decreased antimicrobial activity was detected for the PE testing samples towards both bacteria since MIC and MBC values were higher than those of the DES and SF samples. In that case, these values were above 500 µL for PE sample/mL, the highest concentration tested, corresponding to 148 and 76 µg HA/mL for PE_LA:F_ and PE_LA:U_, respectively. These results suggest that DES testing samples are indeed the most promising compounds to be used for antimicrobial effect against the 2 selected bacterial strains, as their MIC and MBC values ranged from 4 to 16 mg/mL. Previous studies demonstrated that lactic acid, fructose, and urea were shown to have an antimicrobial effect. For instance, lactic acid was proven to be an effective compound for the treatment of both *S. aureus* and *P. aeruginosa* [55]. Similarly, fructose was proven to enhance the efficiency of antibiotics in the treatment *S. aureus*, and its administration in an adjunctive therapy treated *P. aeruginosa* infection [56,57]. Urea also displayed an antibacterial effect mainly on *S. aureus* growth [58]. Furthermore, the DES combinations used in this study could be used as potential preservatives in the formulation as lactic acid has been shown to increase the shelf life of the eye drop [39]. This would eliminate the need for single-use formulations without artificial preservatives. Moreover, the presence of urea could contribute to improvements for dry eye patients, as it was proven to promote the formation of the lipid layer in the tear film [46,47]. Fructose is a natural, non-toxic compound present in the corneal epithelium, at a concentration gradient to the aqueous humor and to the stroma, and could be used for ocular applications when used within safe concentrations [41,42,59].

Regarding the tested commercial eye drops, it was observed that the HA-based commercial eye drops did not show antimicrobial activity against the microorganisms studied. This result is expected since the function of this product is not anti-infective, as HA does not act as an antimicrobial agent and is mainly efficient to maintain ocular hydration and comfort [18,60]. On the other hand, the ophthalmic anti-infective commercial product composed of chloramphenicol was shown to be more efficient against the microorganisms identified in dry eye patients as it is a well-known antimicrobial agent that validates the assays performed.

## 4. Conclusions

The current work presents a process that contributes to the valorization of inedible food waste. A green technique was applied to obtain a HA from TVH using lactic acid-based DES for the valorization of industrial by-products. The DES, SF, and PE testing samples were tested in vitro for their potential application in dry eye disease therapy. A range of concentrations of the samples was studied to analyze the antioxidant and anti-inflammatory response. A higher antioxidant effect was observed for the PE_LA:F_ sample in comparison to the SF_LA:F_ sample, showing a significant decrease of the ROS in comparison to the control. All testing samples did not display a pro-, nor anti-inflammatory response, based on the studied IL-10 and IL-6 levels, except for SF_LA:F_ testing sample at 0.5 mg/mL, which significantly increased IL-6 secretion. Furthermore, DES and SF samples showed antimicrobial activity against dry eye-associated bacteria *S. aureus* and *P. aeruginosa*. In conclusion, DES and SF samples seem to be the most promising samples, and they could be used in ophthalmic therapeutic applications due to their antioxidant and antimicrobial activities. Consequently, the precipitation of the extracts from DES would not be necessary in order to obtain bioactive ingredients, which could reduce processing costs and time for future applications.

## Figures and Tables

**Table 1:** Determination of the minimum inhibitory concentration (MIC) and the minimum bactericidal concentration (MBC) for *S. aureus* and *P. aeruginosa* against the testing samples.

Target Bacteria	Sample ^a^	MIC (µL of Testing Sample/mL)	Sample Composition at MIC Value			MBC (µL of TESTING Sample/mL)	Sample Composition at MBC Value		
			[DES] (mg/mL)	[HA] (ng/mL)	[CHL] (µg/mL)		[DES] (mg/mL)	[HA] (ng/mL)	[CHL] (µg/mL)
*S. aureus *	DES_LA:F_	125	4	—	—	250	8	—	—
	DES_LA:U_	250	8	—	—	500	16	—	—
	SF_LA:F_	250	8	2.30	—	250	8	2.30	—
	SF_LA:U_	250	4	0.67	—	500	16	2.70	—
	PE_LA:F_	>500	—	>148,000	—	>500	—	>148,000	—
	PE_LA:U_	>500	—	>76,000	—	>500	—	>76,000	—
	HA-ED	>500	—	>0.75	—	>500	—	>0.75	—
	CHL-ED	0.98	—	—	7.8	31.25	—	—	250
*P. aeruginosa*	DES_LA:F_	125	4	—	—	125	4	—	—
	DES_LA:U_	125	4	—	—	125	4	—	—
	SF_LA:F_	250	8	2.30	—	250	8	2.30	—
	SF_LA:U_	125	4	0.67	—	250	8	1.30	—
	PE_LA:F_	>500	—	>148,000	—	>500	—	>148,000	—
	PE_LA:U_	>500	—	>76,000	—	>500	—	>76,000	—
	HA-ED	>500	—	>0.75	—	>500	—	>0.75	—
	CHL-ED	15.63	—	—	125	15.63	—	—	125

^a^ Samples abbreviations: Deep eutectic solvents synthesized using lactic acid with fructose and urea are DES_LA:F_ and DES_LA:U_, respectively. The soluble fractions of TVH in DES_LA:F_ and DES_LA:U_ are SF_LA:F_ and SF_LA:U_, respectively. Precipitated extracts from DES_LA:F_ and DES_LA:U_ are PE_LA:F_ and PE_LA:U_, respectively. The commercial eye drops are hyaluronic acid-based eye drop (HA-ED) and chloramphenicol-based eye drop (CHL-ED).

## Data Availability

Not applicable.

## Supplementary Materials

The following supporting information can be downloaded at: https://www.mdpi.com/article/10.3390/foods11030342/s1. Table S1: Concentration of the compounds in the samples studied for antimicrobial analysis. Table S2: Hyaluronic acid, lipids, proteins and ash content in the samples obtained using Deep eutectic solvents synthesized using lactic acid with fructose (DES_LA:F_) and lactic acid with urea (DES_LA:U_). Table S3: Determination of the minimum inhibitory concentration (MIC) and the minimum bactericidal concentration (MBC) for the testing samples against dry eye-associated bacteria *S. aureus* and *P. aeruginosa*, Figure S1: Effect of each compound on TNF-α-induced cells by analyzing IL-10 cytokine release. Deep eutectic solvents synthesized using lactic acid with fructose and urea are DES_LA:F_ and DES_LA:U_, respectively. The soluble fractions of TVH in DES_LA:F_ and DES_LA:U_ are SF_LA:F_ and SF_LA:U_, respectively. Precipitated extracts from DES_LA:F_ and DES_LA:U_ are PE_LA:F_ and PE_LA:U_, respectively. Negative values of IL-10 concentrations are considered experimental deviations due to equipment variation, Figure S2: Effect of each compound on TNF-α-induced cells by analyzing IL-6 cytokine release. Deep eutectic solvents synthesized using lactic acid with fructose and urea are DES_LA:F_ and DES_LA:U_, respectively. The soluble fractions of TVH in DES_LA:F_ and DES_LA:U_ are SF_LA:F_ and SF_LA:U_, respectively. Precipitated extracts from DES_LA:F_ and DES_LA:U_ are PE_LA:F_ and PE_LA:U_, 6 respectively. Negative values of IL-6 concentrations are considered experimental deviations due to equipment variation. ** *p* < 0.01 in comparison to TNF-α-stimulated control cells; + *p* < 0.05, ++ *p* < 0.01, +++ *p* < 0.001 in comparison to non-TNF-α-stimulated cells. References [61,62] are cited in the supplementary materials.
