# Supplementary material for: Potential Ophthalmological Application of Extracts Obtained from Tuna Vitreous Humor Using Lactic Acid-Based Deep Eutectic Systems
Source: Foods. 2022 Jan 25;11(3):342. doi: 10.3390/foods11030342 (PMC8834193; doi:10.3390/foods11030342)
Supplement: Supplementary file 1 [file foods-11-00342-s001.zip › foods-1541264-supplementary.pdf]

Supplementary material

Table S1. Concentration of the compounds in the samples studied for antimicrobial analysis.

	**Composition of the stock solution**					
**Sample [a]**	**[Lactic acid] (mg/mL)**	**[Fructose] (mg/mL)**	**[Urea] (mg/mL)**	**[Extract] (mg/mL)**	**[Hyaluronic acid in extract] (µg/mL)**	**[Chloramphenicol] (mg/mL)**
$DES_{LA:F}$	22.86	9.14	—	—	—	—
$DES_{LA:U}$	27.43	—	4.57	—	—	—
$SF_{LA:F}$	22.80	9.12	—	0.08	10.72	—
$SF_{LA:U}$	27.34	—	4.56	0.10	6.15	—
$PE_{LA:F}$	—	—	—	2.00	0.30	—
$PE_{LA:U}$	—	—	—	2.00	0.15	—
HA-ED [b]	—	—	—	—	1.50	—
CHL-ED [b]	—	—	—	—	—	0.50

[a] Samples abbreviations: Deep eutectic solvents synthesized using lactic acid with fructose and urea are $DES_{LA:F}$ and $DES_{LA:U}$, respectively. The soluble fractions of TVH in $DES_{LA:F}$ and $DES_{LA:U}$ are $SF_{LA:F}$ and $SF_{LA:U}$, respectively. Precipitated extracts from $DES_{LA:F}$ and $DES_{LA:U}$ are $PE_{LA:F}$ and $PE_{LA:U}$, respectively. The commercial eye drops are hyaluronic acid-based eye drop (HA-ED) and chlo-ramphenicol-based eye drop (CHL-ED).
[b] Commercial formulations.

Extract characterization

Hyaluronic acid was analyzed using high-performance liquid chromatography (HPLC), as reported in the Materials and Methods section of the manuscript. The total protein content in the extracts is determined according to Lowry's method [61]. The total lipid content in the extracts is obtained based on the Bligh & Dyer technique [62]. The ash content of the samples was determined by placing them in crucibles and heating them in a high-temperature muffle furnace (Nabertherm, Germany) type LT 15/13, provided with a C450 Controller. The run was conducted for 4 h at a temperature of 550 ºC. The experiments were done in duplicates.

Table S2: Hyaluronic acid, lipids, proteins and ash content in the samples obtained using Deep eutectic solvents synthesized using lactic acid with fructose ($DES_{LA:F}$) and lactic acid with urea ($DES_{LA:U}$).

Concentration in precipitated extract (mg/ 100 mg extract)	$DES_{LA:F}$	$DES_{LA:U}$
Hyaluronic acid	1.99 ± 0.49	1.06 ± 0.22
Lipids	4.97 ± 0.61	5.71 ± 0.98
Proteins	49.66 ± 1.42	50.66 ± 1.67
Ash	27.98 ± 0.65	29.03 ± 0.59

Table S3. Determination of the minimum inhibitory concentration (MIC) and the minimum bactericidal concentration (MBC) for the testing samples against dry eye-associated bacteria *S. aureus* and *P. aeruginosa* [a].

Target Bacteria	Sample [b]	MIC (µL of testing sample/mL)	Sample composition at MIC value			MBC (µL of testing sample/mL)	Sample composition at MBC value		
			[DES] (mg/mL)	[HA] (mg/mL)	[CHL] (mg/mL)		[DES] (mg/mL)	[HA] (mg/mL)	[CHL] (mg/mL)
S. aureus	$DES_{LA:F}$	125 (125/ 125/ 125)	4 (4 / 4 / 4)	—	—	250 (500/ 250/ 250)	8 (16 / 8 / 8)	—	—
	$DES_{LA:U}$	250 (250/ 250/ 250)	8 (8 / 8 / 8)	—	—	500 (250/ 500/ 500)	16 (8 / 16 / 16)	—	—
	$SF_{LA:F}$	250 (250/ 250/ 250)	8 (8 / 8 / 8)	22.7×10^{-4} (22.7×10^{-4}/ 22.7×10^{-4}/ 22.7×10^{-4})	—	250 (250/ 500/ 250)	8 (8 / 16 / 8)	22.7×10^{-4} (22.7×10^{-4}/ 45.5×10^{-4}/ 22.7×10^{-4})	—
	$SF_{LA:U}$	250 (250/ 250/ 250)	4 (4 / 4 / 4)	6.7×10^{-4} (6.7×10^{-4}/ 6.7×10^{-4}/ 6.7×10^{-4})	—	500 (250/ 500/ 500)	16 (8 / 16 / 16)	26.8×10^{-4} (13.4×10^{-4}/ 26.8×10^{-4}/ 26.8×10^{-4})	—
	$PE_{LA:F}$	> 500 (>500/ >500/ >500)	—	> 148 (>148/ >148/ >148)	—	> 500 (>500/ >500/ >500)	—	> 148 (>148/ >148/ >148)	—
	$PE_{LA:U}$	> 500 (>500/ >500/ >500)	—	> 76 (>76/ >76/ >76)	—	> 500 (>500/ >500/ >500)	—	> 76 (>76/ >76/ >76)	—
	HA-ED	> 500	—	> 7.5×10^{-4}	—	> 500	—	> 7.5×10^{-4}	—

		(>500/ >500/ >500)		(7.5×10^{-4}/ 7.5×10^{-4}/ 7.5×10^{-4})		(>500/ >500/ >500)		(7.5×10^{-4}/ 7.5×10^{-4}/ 7.5×10^{-4})	
	CHL-ED	0.98	—	—	7.8 (7.8/ 15.6/ 7.8)	31.25 (62.5/ 31.25/ 31.25)	—	—	250 (313/ 250/ 250)
P. aeruginosa	$DES_{LA:F}$	125 (125/ 125/ 125)	4 (4 / 4 / 4)	—	—	125	4 (4 / 8 / 4)	—	—
	$DES_{LA:U}$	125 (125/ 125/ 125)	4 (4 / 4 / 4)	—	—	125	4 (4 / 4 / 4)	—	—
	$SF_{LA:F}$	250 (125/ 250/ 250)	8 (4 / 8 / 8)	22.7×10^{-4} (22.7×10^{-4}/ 22.7×10^{-4}/ 22.7×10^{-4})	—	250	8 (8 / 8 / 8)	22.7×10^{-4} (22.7×10^{-4}/ 22.7×10^{-4}/ 22.7×10^{-4})	—
	$SF_{LA:U}$	125 (125/ 125/ 125)	4 (4 / 4 / 4)	6.7×10^{-4} (6.7×10^{-4}/ 6.7×10^{-4}/ 6.7×10^{-4})	—	250	8 (4 / 8 / 8)	13.4×10^{-4} (6.7×10^{-4}/ 13.4×10^{-4}/ 13.4×10^{-4})	—
	$PE_{LA:F}$	> 500 (>500/ >500/ >500)	—	> 148 (>148/ >148/ >148)	—	> 500 (>500/ >500/ >500)	—	> 148 (>148/ >148/ >148)	—
	$PE_{LA:U}$	> 500 (>500/ >500/ >500)	—	> 76 (>76/ >76/ >76)	—	> 500 (>500/ >500/ >500)	—	> 76 (>76/ >76/ >76)	—
	HA-ED	> 500 (>500/ >500/ >500)	—	> 7.5×10^{-4} (7.5×10^{-4}/ 7.5×10^{-4}/ 7.5×10^{-4})	—	> 500 (>500/ >500/ >500)	—	> 7.5×10^{-4} (7.5×10^{-4}/ 7.5×10^{-4}/ 7.5×10^{-4})	—
	CHL-ED	15.63 (7.8/ 15.63/ 15.63)	—	—	125 (62.5/ 125/ 125)	15.63 (15.63/ 31.25/ 15.63)	—	—	125 (125/ 250/ 125)

[a] Cells shaded in grey present the concentration with three different runs shown between parentheses of the deep eutectic solvents (DES), hyaluronic acid (HA), and chloramphenicol (CHL) in the testing samples, corresponding to the MIC and MBC values obtained.

[b] Samples abbreviations: Deep eutectic solvents synthesized using lactic acid with fructose and urea are $DES_{LA:F}$ and $DES_{LA:U}$, respectively. The soluble fractions of TVH in $DES_{LA:F}$ and $DES_{LA:U}$ are $SF_{LA:F}$ and $SF_{LA:U}$, respectively. Precipitated extracts from $DES_{LA:F}$ and $DES_{LA:U}$ are $PE_{LA:F}$ and $PE_{LA:U}$, respectively. The commercial eye drops are: hyaluronic acid-based eye drop (HA-ED) and chloramphenicol-based eye drop (CHL-ED).

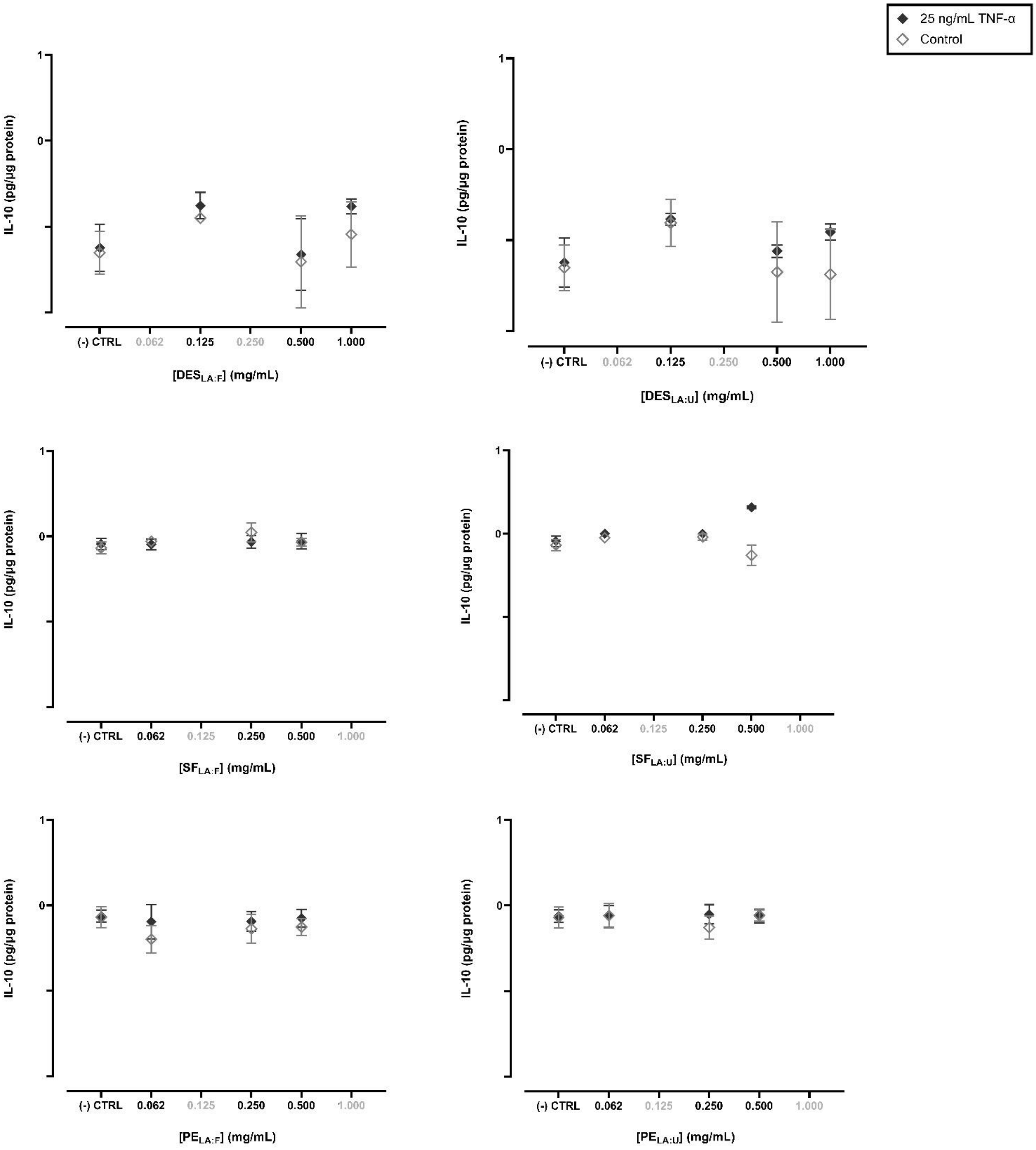

Figure S1. Effect of each compound on TNF-α-induced cells by analyzing IL-10 cytokine release. Deep eutectic solvents synthesized using lactic acid with fructose and urea are DES$_{LA:F}$ and DES$_{LA:U}$, respectively. The soluble fractions of TVH in DES$_{LA:F}$ and DES$_{LA:U}$ are SF$_{LA:F}$ and SF$_{LA:U}$, respectively. Precipitated extracts from DES$_{LA:F}$ and DES$_{LA:U}$ are PE$_{LA:F}$ and PE$_{LA:U}$, respectively. Negative values of IL-10 concentrations are considered experimental deviations due to equipment variation.

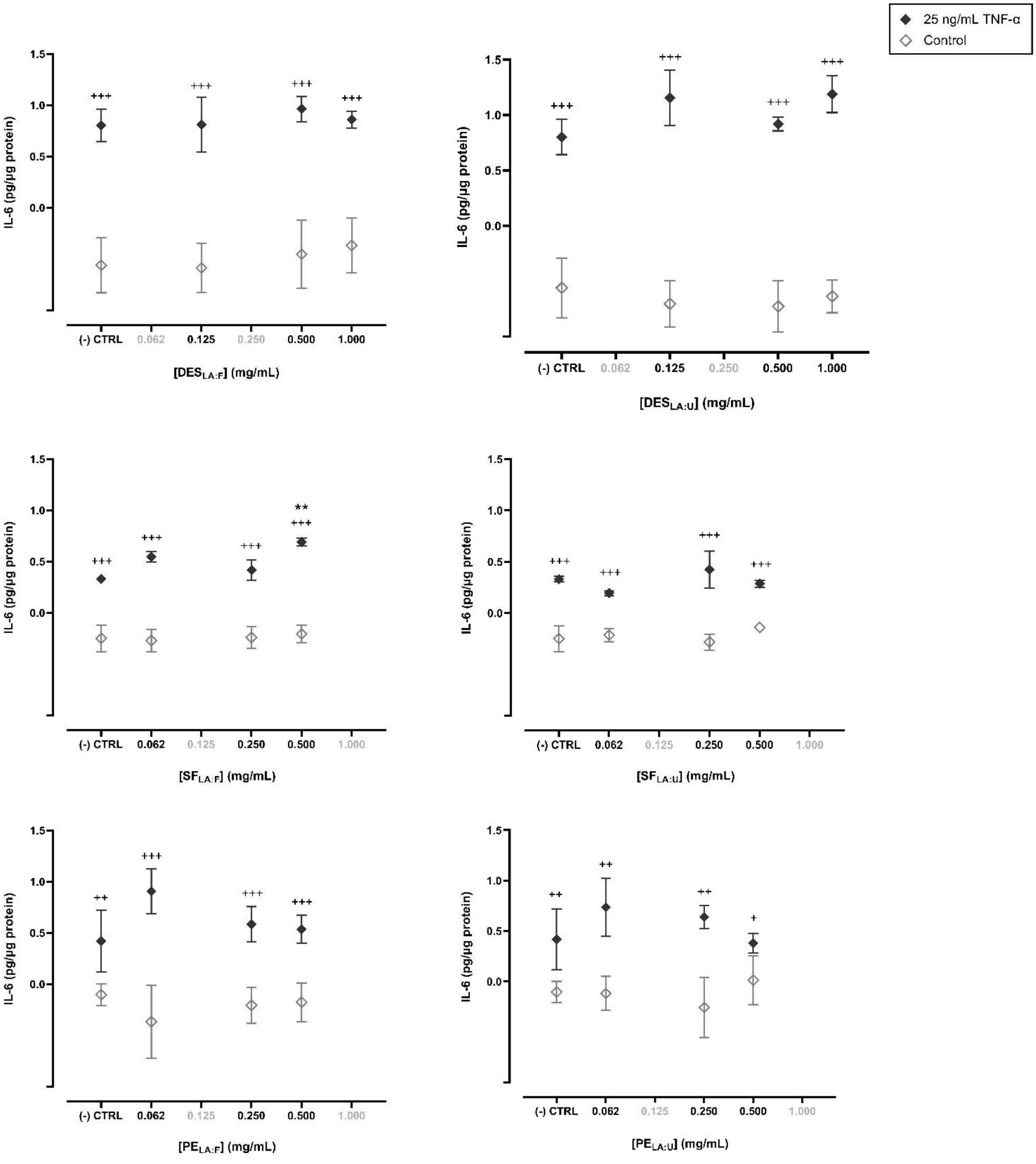

Figure S2. Effect of each compound on TNF-α-induced cells by analyzing IL-6 cytokine release. Deep eutectic solvents synthesized using lactic acid with fructose and urea are $DES_{LA:F}$ and $DES_{LA:U}$, respectively. The soluble fractions of TVH in $DES_{LA:F}$ and $DES_{LA:U}$ are $SF_{LA:F}$ and $SF_{LA:U}$, respectively. Precipitated extracts from $DES_{LA:F}$ and $DES_{LA:U}$ are $PE_{LA:F}$ and $PE_{LA:U}$,

respectively. Negative values of IL-6 concentrations are considered experimental deviations due to equipment variation. **$P < 0.01$ in comparison to TNF-α-stimulated control cells; +$P < 0.05$, ++$P < 0.01$, +++$P < 0.001$ in comparison to non-TNF-α-stimulated cells.
